# The role of cardiac surgery in global surgery and global health: a case study from Tashkent

**DOI:** 10.29392/joghr.3.e2019074

**Published:** 2019-11

**Authors:** Sugy Choi, Dominique Vervoort, Woong-Han Kim

**Affiliations:** 1 Department of Health Law, Policy & Management, Boston University School of Public Health, Boston, Massachusetts, USA,; 2 Program in Global Surgery and Social Change, Harvard Medical School, Boston, Massachusetts, USA,; 3 Program in Global Surgery and Implementation Science, JW Lee Center for Global Medicine, Seoul National University College of Medicine, Seoul, South Korea

**Keywords:** global health

In recent years, surgical care has become an increasingly important part of global health agendas due to its ability to save lives, prevent disability, and promote economic growth.^1^ Cardiovascular diseases (CVDs) have a significant surgical or interventional burden, most notably including ischemic heart disease, congenital heart disease (CHD), rheumatic heart disease, and valvular and aortic conditions. In low- and middle-income countries (LMICs), six billion people lack access to cardiac surgical care when needed. Over one million babies are born annually with CHD, yet over 90% lacks access to timely and appropriate treatment.^2,3^ Nevertheless, over 70% of CHD cases require medical or surgical care within the first year.^4^ If these patients miss the opportunity to receive surgery, they will often die within a year or survive and become severely disabled.

Traditionally, cardiac surgery has been perceived as too expensive, especially for LMIC, where basic surgical infrastructure is often lacking; however, recent data suggests that pediatric cardiac surgery in LMICs is potentially very cost-effective, especially compared to several current global health interventions.^5^ The estimated life expectancy after cardiac surgery and the contribution to society versus the life-long burden of families and society without access to cardiac surgery is significant. Additionally, scaling cardiac surgical services impacts facilities and health systems as a whole due to its cross-cutting and multidisciplinary nature.^6^

## CAPACITY BUILDING

The Program for Global Surgery and Implementation Science at the J.W. Lee Center for Global Medicine, School of Medicine, Seoul National University, was established in 2012 with the aim to pursue the former World Health Organization Director-General, Dr. Jong-Wook Lee’s belief in the importance of for providing health for all.^7^ Since then, the Center has been focusing on reducing health disparities through international cooperation and research. One of the projects in the hospital strengthening unit is the application of a team-based approach to build capacity for pediatric cardiac surgery teams in LMICs. Training teams within the local context has been the core strategy of the program since its start, as a proven cost-effective and sustainable approach to capacity building.^8^ During the surgical training program, high-income country (HIC) (e.g. from South Korea) and LMIC teams (e.g. from Uzbekistan), regardless of their roles, can share their existing knowledge and ideas, and improve their knowledge of cardiac surgery delivery. The program has two major activities: in-country training in a given LMIC (here: Uzbekistan) and an invitational training program in South Korea. For in-country training, participants are embedded in local settings, which allows for sustainable and reality-driven skills development, utilizing the resources, workforce, and health system the facility and providers function in, as opposed to training abroad. However, the training in South Korea facilitates observing management in a tertiary hospital setting, learning new techniques, and keeping up-to-date with the latest technologies.

## TEAM-BASED TRAINING

The HIC team was recruited by the Seoul National University Hospital from 2009 until 2014 and by the J.W. Lee Center for Global Medicine as of 2015. To date, the Center has launched pediatric cardiac surgery capacity building training programs in China, Nepal, Vietnam, Laos, Mongolia, Ethiopia, Cote d’Ivoire, and Uzbekistan. The HIC team included a cardiac surgeon, a pediatric cardiologist, anesthesiologist, perfusionist, intensive care unit (ICU) nurses and administrative assistants to provide comprehensive care, assistance, and capacity building. In Uzbekistan, developing a cardiac surgical team was the first step of these bidirectional institutional partnerships. Communicating the importance of building a cardiac surgery team to enhance the clinical capacity of the workforce in local hospitals was often a compelling argument for hospital leadership. Following a formation of the team with support from the hospital, communication between the visiting team and the local team continued until the annual in-country training began. The in-country training diagnosed and operated on patients in Uzbekistan. Due to a lack of supplies for performing heart surgeries locally, the HIC team imported consumables during every visit and brought enough for local medical practitioners to continue using them for the following six months. Invitational training was conducted bi-annually (2012, 2014, 2016) to increase knowledge of the LMIC team. The invitational training brought a few members of the LMIC team to South Korea (e.g. surgeons and anesthesiologists) to observe and assist surgeries at the Seoul National University Hospital. The LMIC team learned to perform complex surgeries through hands-on training in a HIC and they observed how the HIC teams operate. Education for Uzbekistan medical staff in South Korea emphasized the importance of a team-based approach with small trainees who came to Korea and emphasized the need to build a team after returning to Uzbekistan and continue to educate the whole team.

## ANNUAL IN-COUNTRY TRAINING IN TASHKENT, UZBEKISTAN

Since 2009, on-the-ground capacity building was provided to LMIC team in Tashkent, Uzbekistan. Annually, the Center organizes a volunteer team collaborating with the Tashkent team in a bidirectional nature in order to perform multiple surgeries in a week-long trip and build capacity in the process. Similar to other LMICs, Uzbekistan did not have a training program for cardiothoracic surgeons.^9^ In-country field training consisted of a week-long mission to Tashkent, Uzbekistan, which was conducted by the cardiac surgery team from the Seoul National University Hospital, and transitioned to a team from the J.W. Lee Center for Global Medicine in 2015. The J.W. Lee Centre for Global Medicine provided additional support to arrange and implement the surgical training program in Uzbekistan. For in-country training, the HIC team continued to emphasize the team-based approach, showing and educating that they need to communicate and cooperate towards providing patient-centered care.

During each annual visit, the LMIC and HIC team screened an average of 150 children with probable heart disease. Of the diagnosed cases, around 7–13 patients with the most urgent or severe heart disease were selected for surgical intervention. The selection of cases was jointly made at the end among both teams. The team sent patients with the most challenging conditions to Korea or if additional cardiac catheterization tests were prompted.

A total of 103 patients were operated on together with the visiting team from 2009 to 2018. A variety of surgeries have been performed by both teams and as a result of this training program, the local team learned to conduct large ventricular septal defect (VSD) repair, arterial switch operation, bidirectional cavopulmonary shunt, total correction of tetralogy of Fallot (TOF), total anomalous pulmonary venous return (TAPVR) operation, and coarctation of aorta operation. Led by South Korean cardiothoracic surgeons, the in-country training involved watching the surgery and participating in surgery for LMIC team depending on the case complexity. A case conference was held at the end of the week to reinforce learnings and to share the results to various stakeholders. In between the annual visits, both teams have been communicating through e-mails and phone calls.

Prior to the program’s launch in 2009, a biomedical engineering team from South Korea was also involved in maintaining and configuring operation equipment and facilities. New equipment intended for the operation of infants with complex heart disease was purchased for the facility. A lot of donated equipment tends to be unused in developing countries because recipients do not know how to use or maintain them^10^; however, with yearly visits, the host team was able to learn how to sustain the use of the equipment.

## STAKEHOLDER ENGAGEMENT

In preparing for global cardiac surgery capacity building programs, key stakeholder engagement in LMICs is an important component of a program’s success. The visiting team engaged local charities and the local government to inform planning and the implementation of the training program.

The program was initially funded by the Department of Public Health Medical Service at the Seoul National University Hospital. Since 2014, this program has been funded by the J.W. Lee Center for Global Medicine, and the non-governmental organization Raphael International that received donations from a Korean private company that operated with migrant workers from Uzbekistan. Each year, about US$ 80,000 to US$ 100,000 is budgeted for the program and it was allocated for consumables, shipments, and travel expenses. Donated supplies last at least six months and if financial constraints cannot be overcome, surgeries cannot continue. Funding is a continuing challenge and self-sustainability is an important political agenda that needs to be solved in the near future.

## NEEDS ASSESSMENT

To develop a successful cardiac surgery program, infrastructure and resources are required, including a safe operating space, intensive care unit (ICU), laboratory, wards, and ancillary health services, and a cardiac team consisting of a cardiac surgeon, anesthesiologist, cardiologist, perfusionist, technician, ICU nurse, and supporting personnel. By building the capacity for cardiac surgery, hospitals strengthen all aspects of their care, from pre-hospital care and diagnostics to intra-operative services and post-operative follow-up care. Via post-operative cardiac echocardiography, trained personnel can evaluate the results and anticipate the post-operative course.

## SUSTAINABILITY AND WAY FORWARD

All children survived the operation and in-hospital stay, but no comprehensive data on follow-up was available due to costs of tracking patients, costs of traveling to the hospital for patients, and frequent changes in caregivers’ contact information. Interpreting outcome data should be interpreted with the inherent limitation of the data. The program is starting to track peri-operative mortality and complications starting in 2019. Hosting a team-based training program in a high-income country may be a way to increase effectiveness and sustainability. The HIC team has implemented biannual invitational training programs in South Korea, in addition to the annual visits. Surgeons in LMICs have a strong desire to learn and experience surgical techniques through educational programs from high-income countries.^11^ Team members have mentioned that these learning opportunities are one of the motivators and they helped them to continue to stay at their jobs. In addition to sustaining the surgical improvements, improving nurses’ capacity is crucial to advance pre-, intra-, and post-operative care and the medical environment in LMICs; to support nurses’ critical involvement, the team-based approach importantly includes training for nurses as well. Team members can be cooperative and collaborative by communicating with one another and learning from one another. Training with team members can reinforce their learning and facilitate acceptance to changes in an institutional arrangement. As a team, they can facilitate the implementation of their learnings when they return to their home countries.^12^ Implementing a team-based global surgery program demonstrates support from the institutions, and therefore, such international partnerships can facilitate immediate systematic and organizational changes in the surgery department, which can be an impetus for a change and impact.

After the implementation of the program, organizational and environmental changes occurred in the Tashkent cardiac center. There were ripple effects in the culture of the organization after the noticeable recovery of patients. The biggest stimulus for the cardiac team and the hospital staff included leadership, which altogether led to the sustainability of cardiac surgery and the number of cardiac operations increased substantially in Tashkent. This increase in the number of operations is due to the increase in surgical capacities (eg, TOF and single ventricle) as well as the availability of consumables. Additionally, the budget has been decreasing as fewer medical personnel from the visiting team attended the on-site surgeries as the surgical capacity of the host team has increased.

## CONCLUSION

In the modern era of rapid global advancements in healthcare and bidirectional partnerships to strengthen healthcare services around the world in synergy, acknowledgment of under-addressed, but significantly burdensome diseases such as cardiovascular surgical conditions is critical. This case study highlights how a cardiac surgery program can lead and accelerate improved access to quality surgical care in LMICs, building on a comprehensive strengthening of all services including ICU care and placing team-based capacity-building at its core. Overall, the LMIC team had low turnover rates over the past decade, and have trained together as a team. It is one of the reasons that the LMIC team was able to maintain high medical standards. The medical staffs from the HIC team experienced unintended benefits from the program as well. Feedback from the HIC team revealed positive attitudes toward global health, increased motivation, and improved communication skills. For sustainability of the surgical training programs in LMICs, more research, long-term partnerships,^13^ and evidence-based advocacy is critical. Future studies should consider a rigorous evaluation for surgical training programs in LMICs and create tools to improve sustainability over time.
